# Do family physicians develop ambiguity tolerance as they gain experience? A multicenter cross‐sectional study

**DOI:** 10.1002/jgf2.778

**Published:** 2025-02-03

**Authors:** Hirohisa Fujikawa, Takuya Aoki, Takayuki Ando, Junji Haruta

**Affiliations:** ^1^ Center for General Medicine Education, School of Medicine Keio University Shinjuku‐ku Tokyo Japan; ^2^ Department of Medical Education Studies, International Research Center for Medical Education, Graduate School of Medicine The University of Tokyo Bunkyo‐ku Tokyo Japan; ^3^ Division of Clinical Epidemiology The Jikei University School of Medicine Minato‐ku Tokyo Japan; ^4^ Section of Clinical Epidemiology, Department of Community Medicine, Graduate School of Medicine Kyoto University Sakyo‐ku Kyoto Japan; ^5^ Medical Education Center, School of Medicine Keio University Shinjuku‐ku Tokyo Japan

**Keywords:** ambiguity tolerance, burnout, clinical context, family physicians, tolerance for ambiguity, tolerance of ambiguity

## Abstract

**Background:**

Ambiguity tolerance is important because it contributes to both better patient care and physician well‐being. Although a recent study showed that family physicians have greater ambiguity tolerance than non‐family physicians, the question of when family physicians develop tolerance remains unanswered. Thus, the aim of this study was to examine the associations between the number of postgraduate years (PGYs) and ambiguity tolerance.

**Methods:**

This was a nationwide cross‐sectional study involving family physicians in 14 residency programs throughout Japan. Ambiguity tolerance was assessed as the primary outcome using the Japanese version of the Tolerance for Ambiguity in Medical Students and Doctors scale. Secondary outcomes were burnout and work engagement, assessed using the Japanese version of the Burnout Assessment Tool and the ultra‐short version of the Utrecht Work Engagement Scale, respectively.

**Results:**

173 family physicians were included in the analysis. Physicians of PGY ≥7 had significantly greater ambiguity tolerance and lower burnout risk than those of PGY 3–6. Physicians of PGY 7–20 had significantly higher work engagement than those of PGY 3–6.

**Conclusions:**

Family medicine resident physicians may be vulnerable for the duration of the residency program, although they may develop ambiguity tolerance and improve well‐being over the course of the program. Supervisors in family medicine residency programs should examine the learning environment of their program, considering the vulnerability of their trainees.

## 1 INTRODUCTION

Ambiguity, defined as a lack of reliable, credible, or adequate information,^1^ has been a part of medicine since its inception. As the landscape of healthcare delivery is changing rapidly, physicians are faced with the need to deal with more and different kinds of ambiguity.^2^ For example, physicians have to manage ambiguities in diagnosis and ambiguous therapeutic outcome issues.^3^ Accordingly, tolerance for ambiguity has attracted substantial attention in the medical education literature as a crucial competency for physicians.^4^, ^5^ Lower ambiguity tolerance can impair the quality of patient care. Decreased tolerance for ambiguity of physicians may be associated with more negative attitudes toward underserved patients and increased ordering of diagnostic tests.^6^, ^7^ A positive link may exist between ambiguity tolerance and empathy toward patients.^8^ In addition, tolerance of ambiguity also contributes to the well‐being of physicians themselves: a recent systematic review showed a positive relationship between tolerance and psychological well‐being among physicians,^9^ while a recent study revealed that higher ambiguity tolerance in the clinical context is associated with lower burnout and higher work engagement.^10^ Thus, physician tolerance of ambiguity is a critical need that should be fostered.

Among physicians, family physicians in particular seem to be expected to have high ambiguity tolerance.^11^ Reasons include their frequent engagement with patients with undifferentiated problems and with various systemic issues and work in institutions with limited access to diagnostic testing.^11^, ^12^, ^13^ In fact, a recent Japanese study found that family physicians had significantly greater clinical context‐specific ambiguity tolerance than non‐family physicians.^14^ This finding suggests that tolerance for ambiguity is essential among family physicians and that it is desirable that they develop it.

Against this background, the following question arises: when do family physicians develop their tolerance for ambiguity? Many hypotheses to explain this question have been proposed: in the long journey as a family physician, tolerance for ambiguity might accelerate; ambiguity tolerance might develop during the family medicine residency; ambiguity tolerance might peak at the time of residency graduation and not change thereafter; and family physicians might already have high ambiguity tolerance at the time they initially enter the residency program, which remains largely unchanged thereafter. Given that lower ambiguity tolerance can lead to higher burnout risk and lower work engagement,^10^ we considered that verifying the associations between postgraduate year (PGY), tolerance for ambiguity, burnout, and work engagement is of high priority. To our knowledge, however, no studies have examined these hypotheses.

Here, to better understand the associations of PGY among family physicians with ambiguity tolerance and its related factors of burnout and work engagement, we conducted a multicentered cross‐sectional study.

## 2 METHODS

### 2.1 Study design

This multicentered cross‐sectional study was conducted in February 2024 in Japan as part of a research project exploring physician tolerance for ambiguity and related factors.

### 2.2 Study participants

Family physicians in Japan are known by various names, including general medicine physicians and general practitioners, in addition to family physicians, and these terms are frequently used interchangeably.^15^ In this study, FPs were defined as “Japan Primary Care Association (JPCA)‐certified family physicians, JPCA‐certified primary care physicians, Japanese Medical Specialty Board (JMSB)‐certified general medicine specialists, or residents in these specialties.” The JPCA is the recognized certifying body for Japanese primary care physicians.^16^, ^17^ Its training system for family physicians has been given international certification from the World Organization of National Colleges, Academies and Academic Associations of General Practitioners/Family Physicians.^18^ The JMSB aims to certify specialists independently of academic and scientific perspectives.^19^, ^20^ In 2018, the JMSB designated general medicine as one of the basic specialties. Given that these physicians work flexibly to meet the needs of their institutions, perform a variety of tasks, and attempt to provide comprehensive patient care, we decided to define them all as family physicians in this study.

After graduation from medical school and obtaining a medical license, physicians in Japan rotate through several clinical departments on a monthly basis for the first 2 years (i.e., junior residency program). After the 2 years of training, they proceed to specialty training (i.e., senior residency program) in the department of their choice.^21^, ^22^ Given the circumstances of Japan's postgraduate medical education system described here, we included physicians at or above PGY 3.

### 2.3 Data collection

We recruited 14 residency program directors of family medicine who expressed willingness to cooperate with our study. The programs varied in size and location. The directors were asked to distribute our online questionnaire on SurveyMonkey (www.surveymonkey.com) to their program members.

Before participating in the study, participants read a description of the study and checked a consent box to indicate their agreement. Only those who consented were able to complete the anonymous, self‐administered questionnaire.

### 2.4 Measurements

#### 2.4.1 Primary outcome: Ambiguity tolerance in the clinical context

We assessed tolerance of ambiguity in the clinical context using the Japanese version of the Tolerance for Ambiguity in Medical Students and Doctors (J‐TAMSAD) scale.^3^, ^23^ Previous research has confirmed the good reliability and validity of this scale.^23^ It has 18 items, each rated on a 5‐point Likert scale from 1 = strongly disagree to 5 = strongly agree. The scale score is calculated using the following formula: J‐TAMSAD total score = 25*(average score of the 18 items − 1). The score ranges from 0 to 100, with higher scores indicating a greater tolerance for ambiguity specific to the clinical context.^23^

#### 2.4.2 Secondary outcome: Burnout and work engagement

Burnout, a condition resulting from chronic work‐related stress that has not been successfully managed, is prevalent among physicians and of growing international concern.^24^, ^25^ In this study, burnout was measured using the Japanese version of the Burnout Assessment Tool (BAT‐J).^26^, ^27^, ^28^ Previous research has confirmed the good reliability and validity of this scale.^26^ It has 23 items, each rated on a 5‐point Likert scale, ranging from 1 = never to 5 = always. The BAT‐J score is the average of the 23 items, with higher scores indicating greater risk of burnout.^26^

Work engagement is not the opposite of Burnout, but a personal aspect that complements burnout.^29^, ^30^ It is defined as a positive and fulfilling state of mind related to work that is characterized by vigor, dedication, and absorption.^31^ Workers with a high level of work engagement are full of energy (vigor), highly involved in their work, and are frequently focused and willingly immersed in their work.^31^ In the present study, we evaluated work engagement using the ultra‐short version of the Utrecht Work Engagement Scale (UWES), which has good reliability and validity.^32^, ^33^ The scale is composed of three items that are rated on a seven‐point Likert scale: 0 = never to 6 = always. The score is calculated by averaging the responses for all three items and ranges from 0 to 6, with greater scores meaning higher work engagement.

#### 2.4.3 Independent variable: PGYs

We included PGY as an independent variable, categorized into the four categories of 3–6 (reference category), 7–10, 11–20, and ≥21. This categorization was based on quartiles and the fact that PGY 3–6 corresponds to senior residency status in Japan.

### 2.5 Statistical analysis

First, descriptive statistics were performed on the data in our sample. We reported continuous data as means and deviations and categorical variables as frequencies and percentages. We also calculated the Pearson correlation coefficients between the J‐TAMSAD scale scores, the BAT‐J scores, and the UWES scores. The absolute value of the correlation coefficient was considered meaningful if it was above 0.30.^34^

We then checked for a clustering effect by calculating the intraclass correlation coefficient.^35^ In our dataset, the intraclass correlation coefficient was below 0.1 for the outcome variables (J‐TAMSAD scale, BAT‐J, and UWES). Accordingly, because the clustering effect was considered small,^35^ we performed multivariable linear regression analysis. Prior to analysis, we checked to make sure that the assumptions of residual normality and equal variance were met. Our regression model was adjusted for gender because of its potential association with ambiguity tolerance, burnout, and work engagement.^36^, ^37^

Given that the required sample size per number of independent variables was >20^38^ and the number of independent variables was 4, we estimated a minimum sample size of 80. Because of the small percentage of participants with missing data, we chose complete case analysis. Statistical analyses were conducted using SPSS Statistics version 29.0 (IBM Corp), and a two‐sided *p* < 0.05 was considered to indicate statistical significance.

## 3 RESULTS

Of the 651 eligible participants, 178 (27.3%) completed the survey. Of these, 5 respondents had missing data and were excluded, and the remaining 173 respondents were included in the analysis. Participant characteristics are shown in Table 1. The most prevalent PGY group was 11–20 (56, 32.4%), followed by 3–6 (53, 30.6%). We show the results of the correlation coefficients in the Supporting Infomation.

**TABLE 1:** Characteristics of the participants (*N* = 173).

	Value
Gender, *n* (%)	
Female	59 (34.1)
Male	114 (65.9)
PGYs, *n* (%)	
3–6	53 (30.6)
7–10	36 (20.8)
11–20	56 (32.4)
≥21	28 (16.2)
J‐TAMSAD scale scores^a^, mean (SD)	56.55 (10.60)
BAT‐J scores^b^, mean (SD)	2.44 (0.57)
UWES scores^c^, mean (SD)	3.37 (1.10)

Abbreviations: BAT‐J, Japanese version of the Burnout Assessment Scale; J‐TAMSAD, Japanese version of the Tolerance of Ambiguity in Medical Students and Doctors; PGY, postgraduate year; SD, standard deviation; UWES, Utrecht Work Engagement Scale.

^a^ Ranging from 0 to 100, with higher scores indicating a higher ambiguity tolerance.

^b^ Ranging from 1 to 5, with higher scores indicating a higher burnout risk.

^c^ Ranging from 0 to 6, with higher scores indicating higher level of work engagement.

In Table 2, we show the results of multivariable linear regression analyses of the associations between PGYs, ambiguity tolerance, burnout, and work engagement. After adjusting for gender, physicians of PGY 7–10, 11–20, and ≥21 had significantly higher ambiguity tolerance than those of PGY 3–6 (PGY 7–10, adjusted mean difference 6.68, 95% confidence interval (CI) 2.33 to 11.03; PGY 11–20, adjusted mean difference 6.20, 95% CI 2.33 to 10.08; PGY ≥21, adjusted mean difference 5.99, 95% confidence interval (CI) 1.26 to 10.72). Physicians of PGY ≥7 also had significantly lower risk of burnout than those of PGY 3–6 (PGY 7–10, adjusted mean difference −0.50, 95% CI −0.72 to −0.28; PGY 11–20, adjusted mean difference −0.50, 95% CI −0.70 to −0.31; PGY ≥21, adjusted mean difference −0.58, 95% CI −0.82 to −0.34). While physicians of PGY 7–10 and 11–20 also had greater work engagement than those of PGY 3–6 (PGY 7–10, adjusted mean difference 0.67, 95% CI 0.21 to 1.13; PGY 11–20, adjusted mean difference 0.57, 95% CI 0.16 to 0.97), the UWES scores of physicians of PGY ≥21 were not significantly higher than those of physicians of PGY 3–6.

**TABLE 2:** Associations between PGY, J‐TAMSAD scale score, BAT‐J score, and UWES score (*N* = 173).

	Unadjusted mean difference (95% CI)	Adjusted^a^ mean difference (95% CI)
J‐TAMSAD scale^b^		
PGYs		
3–6	Ref.	Ref.
7–10	6.54 (2.15 to 10.93)**	6.68 (2.33 to 11.03)**
11–20	5.83 (1.94 to 9.73)**	6.20 (2.33 to 10.08)**
≥21	6.48 (1.73 to 11.23)**	5.99 (1.26 to 10.72)*
BAT‐J^c^		
PGYs		
3–6	Ref.	Ref.
7–10	−0.50 (−0.72 to −0.28)**	−0.50 (−0.72 to −0.28)**
11–20	−0.50 (−0.70 to −0.31)**	−0.50 (−0.70 to −0.31)**
≥21	−0.58 (−0.82 to −0.34)**	−0.58 (−0.82 to −0.34)**
UWES^d^		
PGYs		
3–6	Ref.	Ref.
7–10	0.66 (0.20 to 1.12)**	0.67 (0.21 to 1.13)**
11–20	0.53 (0.12 to 0.94)*	0.57 (0.16 to 0.97)**
≥21	0.54 (0.04 to 1.04)*	0.49 (−0.00 to 0.99)

Abbreviations: BAT‐J, Japanese version of the Burnout Assessment Scale; CI, confidence interval; J‐TAMSAD, Japanese version of the Tolerance of Ambiguity in Medical Students and Doctors; PGY, postgraduate year; UWES, Utrecht Work Engagement Scale.

^a^ Adjusted for gender.

^b^ Scores range from 0 to 100.

^c^ Scores range from 1 to 5.

^d^ Scores range from 0 to 6.

*p < 0.05; **p < 0.01.

## 4 DISCUSSION

This nationwide cross‐sectional study focused among family physicians in Japan verified the associations between PGY, ambiguity tolerance, burnout, and work engagement. Physicians who completed a family medicine residency had higher ambiguity tolerance, lower burnout risk, and higher work engagement than those who were still in family medicine residency. In addition, the study indicated that tolerance for ambiguity and its related factors were unlikely to change significantly after residency graduation. Our study provides supervising physicians with insights into family medicine residents' ambiguity tolerance and related vulnerabilities and offers them the opportunity to evaluate and improve their program's learning environment.

To our knowledge, this is the first study to examine the association between PGY and ambiguity tolerance, making our findings a valuable addition to the understanding of postgraduate medical education and the development of ambiguity tolerance. As noted in the Introduction, a recent study found that family physicians demonstrated significantly higher tolerance for ambiguity than non‐family physicians.^14^ This finding in turn suggests that tolerance for ambiguity is a crucial attribute for family physicians. Our study supports the possibility that family medicine training may foster the ambiguity tolerance of resident physicians. Although the mechanisms are unclear, we suggest the following two reasons. First, as noted, family physicians and their trainees frequently encounter various ambiguities, such as undifferentiated patient issues and limited resources.^11^, ^12^, ^13^ These exposures may result in the gradual development of tolerance for ambiguity among family medicine residents. In Japan, the rapid increase in exposure to clinical ambiguity typically occurs from PGY 3 onward. We speculate that the relatively few decision‐making opportunities provided in medical school and early residency might contribute to the development of ambiguity tolerance in the later stages of residency. Second, the presence of experienced supervisors might promote ambiguity tolerance for structured reflection among residents. Regular mentor‐mentee reflection, a common practice in Japanese residency programs,^39^ may contribute to this growth. Further research, particularly qualitative research, would help clarify how ambiguity tolerance develops among family physicians. It also remains unclear whether a similar relationship between PGY and ambiguity tolerance exists among non‐family physicians, rendering this also a valuable area for future study.

Of note, the association we identified between PGY, burnout, and work engagement highlights significant findings that have not been previously identified. One possible explanation is that lower ambiguity tolerance may contribute to increased burnout risk and reduced work engagement,^10^ given that physicians with lower ambiguity tolerance feel more psychological stress, leading to burnout and diminished engagement.^9^, ^40^ Another possible explanation is that the intensity of senior residency programs, where trainees face frequent rotations through different medical institutions and departments, often every several months, along with an increase in clinical responsibilities from PGY 3 onward. Such rapid change in the educational environment may contribute to higher burnout risk and lower work engagement among PGY 3–6 physicians compared to PGY ≥7 physicians. Thus, program supervisors should assess the learning environment and strive to help residents build tolerance for ambiguity, reduce burnout risk, and enhance work engagement.

The study also indicated that tolerance for ambiguity plateaus after completion of the family medicine residency. Although the precise mechanisms are unclear, we suggest two possible explanations. First, after gaining sufficient practical experience during residency, daily clinical practice may become more routine, albeit that this appears unlikely to fully account for the finding given the persistence of ambiguity in medical practice. Second, residents may reach an upper threshold of tolerance for ambiguity during residency, in which the plateau marks the educational impact of their training.

The finding that the UWES scores of PGY ≥21 physicians were not significantly higher than PGY 3–6 physicians is surprising. As we described in the Methods section, work engagement is not the opposite of burnout; rather, it is a personal aspect that complements burnout. This may be why the results for burnout and work engagement showed different trends. However, since the mechanism has not been elucidated, further research, particularly qualitative research involving interviews with relevant individuals, would be insightful.

There are some potential limitations in our study. First, it was conducted under a cross‐sectional design, and the causality or direction of the associations between PGYs and the outcome variables cannot be determined. Confirmation will require studies with a longitudinal design. Second, the response rate was relatively low, which may raise concerns of selection bias. Physicians with lower ambiguity tolerance, higher burnout risk, or less work engagement were possibly less likely to complete the questionnaire. Additionally, given that the Japanese medical system is supported by the self‐sacrificing work of physicians, particularly among those in their 20s and 30s,^22^, ^41^ younger participants were likely to be more occupied and have less time to complete the survey. Future studies should try to maximize the response rate. Third, we may not have been able to control for unknown confounding factors. Fourth, the possibility of social desirability bias cannot be excluded. It is possible that participants responded in a manner that resulted in higher ambiguity tolerance scale scores, lower burnout scale scores, and higher work engagement scale scores. However, we used the methods to minimize this concern (e.g., anonymous survey design). Fifth, while this study showed statistically significant associations between PGY, ambiguity tolerance, burnout, and work engagement, the clinical significance of these associations remains unclear. Additional studies to verify the interpretability of the scales (i.e., J‐TAMSAD scale, BAT‐J, and UWES) could enhance the significance of the study.

## 5 CONCLUSIONS

The study examined differences in ambiguity tolerance, burnout risk, and work engagement between family physicians of PGY 3–6 and those of PGY 7 or above. Trainees may be vulnerable for the duration of their family medicine residency program, although they may develop tolerance for ambiguity and improve their well‐being over the course of the program. Our findings provide family medicine supervisors with additional insight into the ambiguity tolerance of trainees and related factors and, in turn, offer them the opportunity to review the learning environment of their respective programs.

## AUTHOR CONTRIBUTIONS

**Hirohisa Fujikawa:** Conceptualization; investigation; funding acquisition; writing – original draft; methodology; software; formal analysis; project administration; data curation. **Takuya Aoki:** Conceptualization; methodology; writing – review and editing; software; formal analysis; supervision; investigation. **Takayuki Ando:** Conceptualization; methodology; writing – review and editing; supervision; investigation. **Junji Haruta:** Conceptualization; methodology; writing – review and editing; investigation; supervision.

## FUNDING INFORMATION

This work was supported by the Pfizer Health Research Foundation, Japan (grant no. 23‐Y‐13), and the Japan Society for the Promotion of Science, Japan (grant no. JP23K19809).

## CONFLICT OF INTEREST STATEMENT

The authors declare no conflict of interest.

## ETHICS APPROVAL STATEMENT

This study was approved by the ethical committee of Keio University School of Medicine (no. 20231161).

## PATIENT CONSENT STATEMENT

Prior to participating in the study, all participants were asked to read an explanation of the study and provide informed consent by checking the consent box.

## CLINICAL TRIAL REGISTRATION

None.

## Supporting information


Appendix S1.

## Data Availability

The corresponding author may, upon reasonable request, provide the data sets generated and analyzed in the study.

## References

[1] Hillen MA , Gutheil CM , Strout TD , Smets EMA , Han PKJ . Tolerance of uncertainty: conceptual analysis, integrative model, and implications for healthcare. Soc Sci Med. 2017;180(1):62–75. 28324792 10.1016/j.socscimed.2017.03.024

[2] Maini A , Saravanan Y , Singh TA , Fyfe M . Coaching skills for medical education in a VUCA world. Med Teach. 2020;42(11):1308–1309. 32657666 10.1080/0142159X.2020.1788713

[3] Hancock J , Roberts M , Monrouxe L , Mattick K . Medical student and junior doctors' tolerance of ambiguity: development of a new scale. Adv Health Sci Educ Theory Pract. 2015;20(1):113–130. 24841480 10.1007/s10459-014-9510-z

[4] Geller G , Grbic D , Andolsek KM , Caulfield M , Roskovensky L . Tolerance for ambiguity among medical students: patterns of change during medical school and their implications for professional development. Acad Med. 2021;96(7):1036–1042. 33149092 10.1097/ACM.0000000000003820

[5] Luther VP , Crandall SJ . Commentary: ambiguity and uncertainty: neglected elements of medical education curricula? Acad Med. 2011;86(7):799–800. 21715991 10.1097/ACM.0b013e31821da915

[6] Carney PA , Yi JP , Abraham LA , Miglioretti DL , Aiello EJ , Gerrity MS , et al. Reactions to uncertainty and the accuracy of diagnostic mammography. J Gen Intern Med. 2007;22(2):234–241. 17356992 10.1007/s11606-006-0036-9 PMC1824735

[7] Ghosh AK . On the challenges of using evidence‐based information: the role of clinical uncertainty. J Lab Clin Med. 2004;144(2):60–64. 15322499 10.1016/j.lab.2004.05.013

[8] Fujikawa H , Aoki T , Son D , Hayashi M , Eto M . Association between tolerance for ambiguity specific to the clinical context and empathy in medical trainees: a multicenter cross‐sectional study in Japan. Med Teach. 2024;46(4):512–518. 37734453 10.1080/0142159X.2023.2259065

[9] Hancock J , Mattick K . Tolerance of ambiguity and psychological well‐being in medical training: a systematic review. Med Educ. 2020;54(2):125–137. 31867801 10.1111/medu.14031 PMC7003828

[10] Fujikawa H , Aoki T , Ando T , Haruta J . Associations of clinical context‐specific ambiguity tolerance with burnout and work engagement among Japanese physicians: a nationwide cross‐sectional study. BMC Med Educ. 2024;24(1):660. 38877544 10.1186/s12909-024-05644-3 PMC11179221

[11] Turner N . Family physicians: first point of contact, last line of defence. Can Fam Physician. 2023;69(7):490–491. 37451998 10.46747/cfp.6907490 PMC10348802

[12] Hellenberg D , Williams FR , Kubendra M , Kaimal RS . Strengths and limitations of a family physician. J Family Med Prim Care. 2018;7(2):284–287. 30090765 10.4103/jfmpc.jfmpc_297_17 PMC6060944

[13] O'Riordan M , Dahinden A , Akturk Z , Ortiz JM , Dağdeviren N , Elwyn G , et al. Dealing with uncertainty in general practice: an essential skill for the general practitioner. Qual Prim Care. 2011;19(3):175–181. 21781433

[14] Fujikawa H , Aoki T , Ando T , Haruta J . Family physicians have greater ambiguity tolerance in the clinical context: a nationwide cross‐sectional study. J Gen Fam Med. 2024;1–7. 10.1002/jgf2.747 PMC11890053 40061390

[15] Yamamoto Y , Haruta J , Goto R , Maeno T . What kinds of work do Japanese primary care physicians who derive greater positive meaning from work engage in? A cross‐sectional study. J Gen Fam Med. 2023;24(2):94–101. 36909785 10.1002/jgf2.595 PMC10000278

[16] Japan Primary Care Association . Outline of the certification system of the Japan Primary Care Association certified family physicians and primary care physicians. 2023. [Accessed 2024 Oct 29]. Available from: https://www.primary‐care.or.jp/file/pdf_file.php?file=senmonininteiyouko

[17] Japan Primary Care Association . About the association. 2024. [Accessed 2024 October 29]. Available from: https://www.primarycare‐japan.com/about.htm

[18] WONCA . WONCA Accreditation and Certification. 2021. [Accessed 2024 October 29]. Available from: https://www.globalfamilydoctor.com/News/WONCAAccreditationandCertification.aspx

[19] The Japanese Medical Specialty Board . About The Japanese Medical Specialty Board. 2024. [Accessed 2024 October 29]. Available from: https://jbgm.org/

[20] Yokokura Y . Policy address. JMAJ. 2016;59(2–3):55–58. 28299241 PMC5333620

[21] Onishi H . History of Japanese medical education. Korean J Med Educ. 2018;30(4):283–294. 30522257 10.3946/kjme.2018.103 PMC6288623

[22] Fujikawa H , Son D , Eto M . Are residents learners or workers? A historical perspective in Japan. Taps. 2021;6(1):122–124.

[23] Fujikawa H , Son D , Hayashi M , Kondo K , Eto M . Translation, adaptation, and validation of the tolerance of ambiguity in medical students and doctors (TAMSAD) scale for use in Japan. BMC Med Educ. 2023;23(1):405. 37277759 10.1186/s12909-023-04391-1 PMC10240119

[24] Dyrbye LN , Burke SE , Hardeman RR , Herrin J , Wittlin NM , Yeazel M , et al. Association of clinical specialty with symptoms of burnout and career choice regret among US resident physicians. JAMA. 2018;320(11):1114–1130. 30422299 10.1001/jama.2018.12615 PMC6233627

[25] World Health Organization . Burn‐out an occupational phenomenon: International Classification of Diseases. 2019. [Accessed 2024 October 29]. Available from: https://www.who.int/news/item/28‐05‐2019‐burn‐out‐an‐occupational‐phenomenon‐international‐classification‐of‐diseases

[26] Sakakibara K , Shimazu A , Toyama H , Schaufeli WB . Validation of the Japanese version of the burnout assessment tool. Front Psychol. 2020;11:1819. 32849072 10.3389/fpsyg.2020.01819 PMC7431961

[27] Schaufeli WB , De Witte H , Desart S . Manual Burnout Assessment Tool (BAT). Internal report. 2020. [Accessed 2024 October 29]. Available from: https://burnoutassessmenttool.be

[28] Schaufeli WB , Desart S , De Witte H . Burnout assessment tool (BAT)—development, validity, and reliability. Int J Environ Res Public Health. 2020;17(24):9495. 33352940 10.3390/ijerph17249495 PMC7766078

[29] Derbis R , Jasiński AM , Craig T . Work satisfaction, psychological resiliency and sense of coherence as correlates of work engagement. Cogent Psychol. 2018;5(1):1451610.

[30] Schaufeli WB . What is engagement. In: Truss C , Delbridge R , Alfes K , Shantz A , Soane E , editors. Employee engagement in theory and practice. Milton Park, Abingdon, Oxon; New York, NY: Routledge; 2013.

[31] Bakker AB , Schaufeli WB . Work engagement. Wiley encyclopedia of management; 2015. p. 1–5. Hoboken, NJ: John Wiley and Sons, Ltd.

[32] Schaufeli WB , Shimazu A , Hakanen J , Salanova M , De Witte H . An ultra‐short measure for work engagement: the UWES‐3 validation across five countries. Eur J Psychol Assess. 2019;35(4):577–591.

[33] Shimazu A , Schaufeli WB , Kosugi S , Suzuki A , Nashiwa H , Kato A , et al. Work engagement in Japan: validation of the Japanese version of the Utrecht work engagement scale. Appl Psychol. 2008;57(3):510–523.

[34] Cohen J . Statistical power analysis. Curr Dir Psychol Sci. 1992;1(3):98–101.

[35] Katz MH . Multivariable analysis: a practical guide for clinicians and public health researchers. 3rd ed. New York: Cambridge University Press; 2011.

[36] Geller G , Faden RR , Levine DM . Tolerance for ambiguity among medical students: implications for their selection, training and practice. Soc Sci Med. 1990;31(5):619–624. 2218644 10.1016/0277-9536(90)90098-d

[37] Kramer M , Könings KD , Prins JT , van der Heijden FMMA , Heyligers IC . Still higher risk for burnout and low work engagement among female residents after 10 years of demographic feminisation. Med Sci Educ. 2024;34(5):1023–1036. 39450025 10.1007/s40670-024-02084-y PMC11496429

[38] Hair JF , Black WC , Babin BJ , Anderson RE . Multivariate data analysis. 8th ed. Andover: Cengage Learning EMEA; 2019.

[39] Japan Primary Care Association . New family medicine specialist system. [Accessed 2024 Oct 29]. Available from: https://www.shin‐kateiiryo.primary‐care.or.jp/

[40] Iannello P , Mottini A , Tirelli S , Riva S , Antonietti A . Ambiguity and uncertainty tolerance, need for cognition, and their association with stress. A study among Italian practicing physicians. Med Educ Online. 2017;22(1):1270009. 28178917 10.1080/10872981.2016.1270009 PMC5328324

[41] Shibuya K , Unno N . Unpaid doctors in Japanese university hospitals. Lancet. 2019;393(10176):1096–1097. 30824118 10.1016/S0140-6736(19)30472-6

