# Supplementary material for: Do family physicians develop ambiguity tolerance as they gain experience? A multicenter cross‐sectional study
Source: J Gen Fam Med. 2025 Feb 3;26(3):231–7. doi: 10.1002/jgf2.778 (PMC12022422; doi:10.1002/jgf2.778)
Supplement: Supplementary file 1 — Appendix S1. [file JGF2-26-231-s001.pdf]

Supplementary file

Supplementary Table : Correlation matrix of the J-TAMSAD scale scores, the BAT-J scores, and the UWES scores

	BAT-J	UWES
J-TAMSAD scale	-0.37***	0.47***
BAT-J		-0.46***
UWES		

*** $p < 0.001$

Abbreviations: BAT-J, Japanese version of the Burnout Assessment Scale; J-TAMSAD, Japanese version of the Tolerance of Ambiguity in Medical Students and Doctors; UWES, Utrecht Work Engagement Scale.
